# Supplementary material for: Efficient optimization with higher-order Ising machines
Source: Nat Commun. 2023 Sep 27;14:6033. doi: 10.1038/s41467-023-41214-9 (PMC10533504; doi:10.1038/s41467-023-41214-9)
Supplement: Supplementary file 1 — Supplementary Information [file 41467_2023_41214_MOESM1_ESM.pdf]

Efficient Optimization with Higher-Order Ising Machines

Connor Bybee[1], Denis Kleyko[1, 2], Dmitri E. Nikonov[3], Amir Khosrowshahi[1, 3], Bruno A. Olshausen[1], and Friedrich T. Sommer[1, 4]

[1]Redwood Center for Theoretical Neuroscience, University of California, Berkeley, CA
[2]Intelligent Systems Lab, Research Institutes of Sweden, Kista, Sweden
[3]Components Research, Intel, Hillsboro, OR
[4]Neuromorphic Computing Lab, Intel, Santa Clara, CA

Supplementary Material

S1 Time to solution for oscillator Ising machines

To compare higher-order and second-order Ising machines, we measure time to solution (TTS), i.e., the time it takes to reach a given quality level:

$$\mathrm{TTS}_{X\%} = \frac{\mathbb{E}[t_{X\%}]}{p_{X\%}}. \tag{1}$$

Here, $p_{X\%}$ is the probability of success, i.e., of achieving the target quality level of $X\%$. $\mathbb{E}[t_{X\%}]$ is the expected time to reach the solution in successful trials. Thus, $\mathrm{TTS}_{X\%}$ is the approximate time to reach a solution that satisfies greater than or equal to $X\%$ of constraints. Fig. S1 presents the TTS 97%, 98%, 99%, and 100% for higher-order and second-order Ising machines. Note that in all cases, the TTS of higher-order Ising machines is about an order of magnitude lower than for second-order Ising machines.

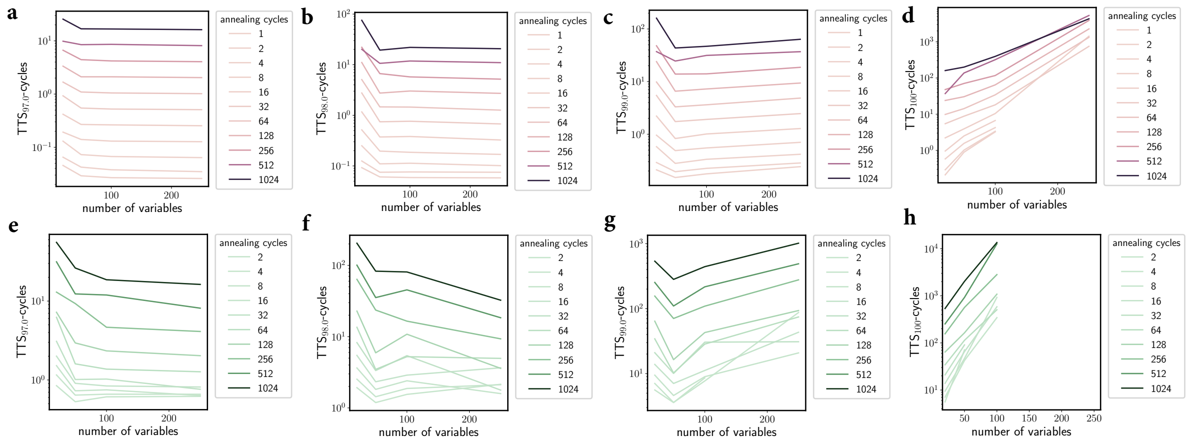

Figure S1: **TTS for higher-order and second-order Ising machines solving the 3SAT benchmark problems.** Each column presents the results for the higher-order model (upper panels **a-d**) and the second-order model (lower panels **e-h**). The four columns correspond to the quality thresholds 97%, 98%, 99%, and 100%, from left to right. Each panel shows results for different problem sizes organized on the horizontal axes. The results for different annealing schedules are displayed by curves with different colors.

S2 Kuramoto versus Hopf oscillator models

In some cases, phase-reduced models of coupled oscillators like the Kuramoto model are sufficient to implement certain computations, e.g., pattern completion in associative memories [1]. However, it has

been shown that coupled oscillator models with both, amplitude and phase dynamics, can outperform phase-reduced models for certain types of computation [2]–[4]. To explore the effects of oscillator properties on solving combinatorial optimization problems, we compared a Kuramoto model and a Hopf model, the latter including phase and amplitude dynamics. The Kuramoto model has one variable per oscillator representing its phase, ϕ_i. The energy function for the h-th constraint for the higher-order Kuramoto oscillator Ising machine used in this study is:

$$E_h(\phi) = \sum_{\mathbf{c}\in\bar{\mathbf{C}}_h} \prod_{i=1}^{k} \Big(1 + \cos(\frac{\pi}{2}(1 - c_i) - \phi_i)\Big)/2. \tag{2}$$

We note that Eq. (2) is similar to the energy function used in [5], the only small difference is that in [5] the energy function is raised to a power of 2. The resulting phase dynamics is:

$$\dot{\phi}_i = \omega_i - r_i(t)\frac{\partial E(\boldsymbol{\phi})}{\partial \phi_i} - q_i(t)\sin(2\phi_i). \tag{3}$$

Here, ω_i is the frequency of the i-th oscillator, $\frac{\partial E(\boldsymbol{\phi})}{\partial \phi_i}$ is the partial derivative of the energy function with respect to the ith oscillator, r_i is the coupling parameter for the i-th oscillator, and q_i is the sub-harmonic injection locking parameter. In our simulations, r_i is held constant, and q_i is annealed linearly as in the Hopf model simulations.
To explore the effect of amplitude dynamics in higher-order Ising models solving SAT problems, we compared the mean energy found by the Kuramoto model to the Hopf model. For each model, a search was performed to find the set of parameters, which minimizes the mean energy. The results in Fig. S2 show that the Hopf model produces solutions with significantly lower mean energies for problem sizes of 50 variables or larger. This empirical comparison motivates our focus on higher-order Ising models with Hopf oscillators in the main manuscript.

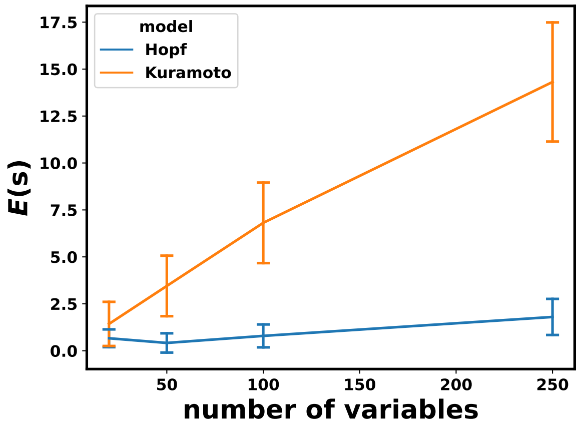

Figure S2: **Hopf versus Kuramoto oscillators.** The mean energy at the end of a trial is plotted against the number of problem variables for benchmark 3SAT problems. The Hopf model (blue) is compared to the Kuramoto model (orange).

S3 Extended simulation results

The parameter configurations that produce the results used in the figures of the main text were found by the grid search. Each parameter value was varied over several orders of magnitude. Parameter configurations with simulation times longer than 1 day were excluded. The simulations were performed on a machine with an NVIDIA 3090 GPU and an 11th Gen Intel Core i9-11900K @ 3.50GHz CPU. The 10 best parameter configurations are reported for each problem size and simulation type in Tables S1-S3. The reported energy and fraction of instances satisfied ("satisfied probability") are averaged 64 over trials for each problem instance and problem instances for a given problem size. The parameter search and optimization in the simulations were done individually for each model, the higher-order Hopf oscillator networks (Table S1), the second-order Hopf oscillator networks (Table S2), and the higher-order Kuramoto networks (Table S3).

# of variables	λ	ρ	r	$q_{\max}$	# of cycles	energy	satisfied probability
20	1.0	-1.0	32.0	32.0	512	0.54	0.61
	2.0	-2.0	64.0	64.0	512	0.67	0.33
			32.0	32.0	1024	0.75	0.38
					512	0.76	0.38
	1.0	-1.0	16.0	16.0	512	0.77	0.38
					1024	0.77	0.38
	4.0	-4.0	1.0	1.0	1024	0.77	0.38
	2.0	-2.0	16.0	16.0	128	0.77	0.38
	1.0	-1.0	1.0	1.0	1024	0.78	0.38
			8.0	8.0	256	0.78	0.38
50	1.0	-1.0	32.0	32.0	1024	0.71	0.44
			16.0	16.0	1024	0.78	0.38
	2.0	-2.0	32.0	32.0	512	0.78	0.38
	1.0	-1.0	8.0	8.0	512	0.81	0.35
	2.0	-2.0	16.0	16.0	256	0.82	0.34
			32.0	32.0	1024	0.83	0.36
	1.0	-1.0	16.0	16.0	512	0.86	0.37
			32.0	32.0	256	0.86	0.35
	2.0	-2.0	32.0	32.0	256	0.86	0.37
					128	0.88	0.33
100	1.0	-1.0	32.0	32.0	1024	1.47	0.19
			8.0	8.0	512	1.48	0.17
	2.0	-2.0	32.0	32.0	256	1.48	0.20
			16.0	16.0	512	1.49	0.16
			32.0	32.0	1024	1.49	0.20
	1.0	-1.0	16.0	16.0	512	1.50	0.20
	2.0	-2.0	16.0	16.0	1024	1.50	0.17
	1.0	-1.0	32.0	32.0	256	1.53	0.16
	2.0	-2.0	16.0	16.0	256	1.54	0.17
	1.0	-1.0	16.0	16.0	256	1.54	0.18
250	1.0	-1.0	32.0	32.0	1024	2.73	0.02
	2.0	-2.0	32.0	32.0	512	2.87	0.01
			16.0	16.0	1024	2.90	0.02
	1.0	-1.0	16.0	16.0	1024	2.91	0.02
	2.0	-2.0	32.0	32.0	1024	2.93	0.03
			16.0	16.0	512	3.07	0.02
			32.0	32.0	256	3.08	0.01
	1.0	-1.0	32.0	32.0	512	3.14	0.02
			16.0	16.0	512	3.15	0.02
			32.0	32.0	256	3.15	0.01

Table S1: **Higher-order oscillator simulation result for top parameter configurations.** The mean energy and fraction of satisfied instances are listed for the top 10 parameter configurations for each problem size obtained from simulations of higher-order oscillator networks.

# of variables	$\Delta E_{\min}$	λ	ρ	r	$q_{\max}$	# of cycles	energy	satisfied probability
20	5	1.0	-1.0	0.10	0.10	1024	0.66	0.51
	10	1.0	-1.0	0.10	0.10	1024	0.66	0.52
	5	1.0	-1.0	0.10	0.10	512	0.67	0.52
	10	1.0	-1.0	0.10	0.10	512	0.71	0.50
	1	1.0	-1.0	1.10	1.10	128	0.77	0.44
				1.05	1.05	128	0.77	0.44
	10	0.5	-0.5	0.10	0.10	1024	0.77	0.47
		2.0	-2.0	0.10	0.10	1024	0.78	0.46
				1.00	1.00	64	0.80	0.40
						128	0.80	0.40
50	10	2.0	-2.0	1.00	1.00	128	1.30	0.20
		0.5	-0.5	0.10	0.10	512	1.37	0.21
		1.0	-1.0	1.00	1.00	64	1.38	0.18
		2.0	-2.0	1.00	1.00	64	1.42	0.20
				0.10	0.10	512	1.44	0.22
		1.0	-1.0	1.00	1.00	128	1.48	0.18
		2.0	-2.0	0.10	0.10	256	1.60	0.15
		1.0	-1.0	0.10	0.10	256	1.60	0.15
	5	1.0	-1.0	1.00	1.00	1024	1.62	0.17
						512	1.66	0.16
100	10	2.0	-2.0	1.00	1.00	128	2.82	0.08
		1.0	-1.0	1.00	1.00	128	3.02	0.05
		2.0	-2.0	1.00	1.00	64	3.02	0.05
		1.0	-1.0	1.00	1.00	64	3.20	0.05
		0.5	-0.5	0.10	0.10	512	3.54	0.02
	5	1.0	-1.0	1.00	1.00	1024	3.59	0.04
						512	3.62	0.04
						256	3.66	0.04
						128	3.69	0.03
		2.0	-2.0	1.00	1.00	64	3.76	0.02
250	10	2.0	-2.0	1.00	1.00	32	9.00	0.00
	5	1.0	-1.0	1.00	1.00	128	9.31	0.00
						512	9.85	0.00
						1024	9.94	0.00
	10	2.0	-2.0	0.10	0.10	1024	10.00	0.00
		0.5	-0.5	0.10	0.10	512	10.02	0.00
	5	1.0	-1.0	1.00	1.00	256	10.27	0.00
	10	2.0	-2.0	1.00	1.00	64	10.50	0.00
		1.0	-1.0	1.00	1.00	64	10.60	0.00
	5	2.0	-2.0	1.00	1.00	64	10.70	0.00

Table S2: **Second-order oscillator simulation result for top parameter configurations.** The mean energy and fraction of satisfied instances are listed for the top 10 parameter configurations for each problem size obtained from simulations of second-order oscillator networks.

# of variables	r	$q_{\max}$	# of cycles	energy	satisfied probability
20	1	0.000	256	1.43	0.25
		0.001	256	1.43	0.25
	10	0.000	16	1.43	0.25
			64	1.43	0.25
		0.001	64	1.43	0.25
		0.010	64	1.43	0.25
	20	0.000	64	1.43	0.25
		0.001	64	1.43	0.25
		0.010	64	1.43	0.25
		0.100	64	1.43	0.25
50	20	0.010	64	3.45	0.00
	50	0.010	64	3.45	0.00
	100	0.001	256	3.45	0.00
			1024	3.45	0.00
		0.010	64	3.45	0.00
			256	3.45	0.00
			1024	3.45	0.00
		0.100	64	3.45	0.00
			1024	3.45	0.00
	10	0.000	64	3.46	0.00
100	100	0.001	256	6.81	0.00
			1024	6.81	0.00
		0.000	1024	6.82	0.00
			256	6.83	0.00
		0.001	64	6.83	0.00
		0.010	256	6.83	0.00
			1024	6.83	0.00
	50	0.000	64	6.84	0.00
	100	0.000	64	6.84	0.00
	50	0.001	64	6.85	0.00
250	50	0.010	64	14.31	0.00
	100	0.001	1024	15.82	0.00
			256	15.84	0.00
		0.010	1024	15.84	0.00
		0.000	1024	15.85	0.00
		0.010	64	15.86	0.00
			256	15.86	0.00
		0.100	1024	15.86	0.00
		0.000	256	15.87	0.00
			64	15.90	0.00

Table S3: **Higher-order Kuramoto oscillator simulation result for top parameter configurations.** The mean energy and fraction of satisfied instances are listed for the top 10 parameter configurations for each problem size obtained from simulations of higher-order Kuramoto oscillator networks.

The Tables S1-S3 can be compared to get a sense of how sensitive the quality of the solution is to changes in the parameter values. In regards to the quality of the solution, across a wider range of parameter values the higher-order Hopf oscillator model (Table S1) tends to find solutions that have lower energy and a greater probability of satisfying the problem instance compared to the second-order Hopf oscillator model (Table S2) and the higher-order Kuramoto oscillator model (Table S3). For the Hopf oscillator models, increasing r and $q_{\max}$ tended to increase the solution quality. Though, increasing r and $q_{\max}$ could result in prohibitively long simulation times. Additionally, the higher-order Hopf and Kuramoto oscillator models tended to be less sensitive to the magnitude of the parameter values compared to the second-order Hopf oscillator model. In future work, a more detailed examination and analysis of the impacts of parameter configurations on the performance of Ising machines may be useful for specifying the parameters of potential hardware systems (e.g., their dynamic range).

Supplementary References

[1] T. Nishikawa, Y.-C. Lai, and F. C. Hoppensteadt, "Capacity of oscillatory associative-memory networks with error-free retrieval," *Physical review letters*, vol. 92, no. 10, p. 108 101, 2004.

[2] L. V. Gambuzza, J. Gómez-Gardeñes, and M. Frasca, "Amplitude dynamics favors synchronization in complex networks," *Scientific reports*, vol. 6, no. 1, p. 24 915, 2016.

[3] M. H. Matheny, J. Emenheiser, W. Fon, A. Chapman, A. Salova, M. Rohden, J. Li, M. Hudoba de Badyn, M. Pósfai, L. Duenas-Osorio, *et al.*, "Exotic states in a simple network of nanoelectromechanical oscillators," *Science*, vol. 363, no. 6431, eaav7932, 2019.

[4] J. H. Woo, C. J. Honey, and J.-Y. Moon, "Phase and amplitude dynamics of coupled oscillator systems on complex networks," *Chaos: An Interdisciplinary Journal of Nonlinear Science*, vol. 30, no. 12, p. 121 102, 2020.

[5] M. K. Bashar, Z. Lin, and N. Shukla, "Formulating oscillator-inspired dynamical systems to solve Boolean satisfiability," *arXiv preprint arXiv:2209.07571*, 2022.
